# A Novel Neural Network Model for Blood Pressure Estimation Using Photoplethesmography without Electrocardiogram

**DOI:** 10.1155/2018/7804243

**Published:** 2018-03-07

**Authors:** Ludi Wang, Wei Zhou, Ying Xing, Xiaoguang Zhou

**Affiliations:** ^1^Automation School, Beijing University of Posts and Telecommunications, Beijing 100876, China; ^2^Functional Pharmacology, Department of Neuroscience, Uppsala University, Uppsala, Sweden

## Abstract

The prevention, evaluation, and treatment of hypertension have attracted increasing attention in recent years. As photoplethysmography (PPG) technology has been widely applied to wearable sensors, the noninvasive estimation of blood pressure (BP) using the PPG method has received considerable interest. In this paper, a method for estimating systolic and diastolic BP based only on a PPG signal is developed. The multitaper method (MTM) is used for feature extraction, and an artificial neural network (ANN) is used for estimation. Compared with previous approaches, the proposed method obtains better accuracy; the mean absolute error is 4.02 ± 2.79 mmHg for systolic BP and 2.27 ± 1.82 mmHg for diastolic BP.

## 1. Introduction

Blood pressure (BP) is the driving force for the flow of blood through the blood vessels and reflects the cardiovascular health of the human body. At present, hypertension is the most significant risk factor for cardiovascular and cerebrovascular diseases, identified by the World Health Organization [1] as the main cause of death and disability among the elderly. Poorly controlled hypertension increases the risk of heart attacks, strokes, kidney failure, and heart failure.

Table 1 lists the classification criteria for hypertension in adults (age > 18 years). A normal BP for an adult human is 120/80 mmHg. A systolic blood pressure (SBP) of between 140 and 159 mmHg or diastolic blood pressure (DBP) of between 90 and 99 mmHg is defined as the first stage of hypertension, while the second stage is when SBP is higher than 159 mmHg, or DBP is higher than 99 mmHg [2].

Invasive BP measurement has the highest accuracy of several methods available for measuring BP, but it is not widely applied because of its difficulty and high risk. Using Korotkoff sounds to estimate SBP and DBP is another auscultatory measurement, and this has been widely accepted as the gold standard [3, 4]. Despite its high degree of accuracy and reliability, the auscultatory method does not apply to home blood pressure measurement (HBPM) [5], as it requires a treained professional [6]. Furthermore, the mercury sphygmomanometer is gradually being removed from clinical use [6]. Oscillometric blood pressure measurement has become increasingly popular in automated blood pressure measurement devices [7]. This method uses an electronic pressure sensor to observe the pressure oscillation in the cuff, during its gradual deflation from above SBP to below DBP. The oscillation amplitude increases to its maximum value when the cuff pressure reaches the mean arterial pressure and then gradually decreases with subsequent deflation of the cuff pressure [8]. However, it cannot provide continuous beat-to-beat BP measurement with its periodic features, and it is not appropriate for home healthcare or easing the workload of clinicians at hospitals.

For cuffless BP measurement, the pulse transit time (PTT) method and photoplethysmography (PPG) are widely used techniques [9]. PTT is defined as the time taken for the arterial pulse pressure wave to travel from the aortic valve to the periphery [10], and some researchers have used it to estimate BP indirectly [11]. However, there are two parameters required to calculate PTT, electrocardiogram (ECG), and PPG. As a result, calculation of PPT commonly requires two devices to obtain these two parameters—the ECG is measured at the wrist or chest, and the PPG is measured from the index finger [12].

**Figure 1:**
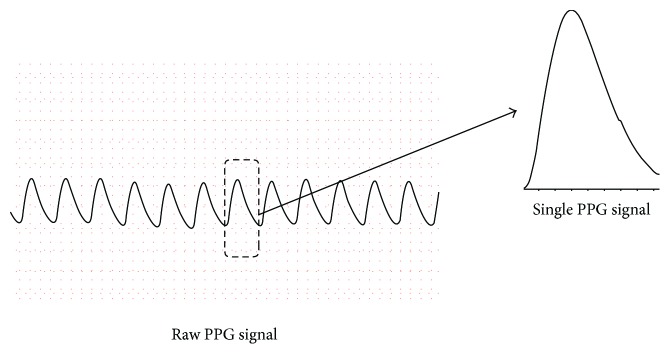
The continuous PPG waveform and one single PPG waveform extracted from it.

Wearable pulse rate sensors based on PPG have become increasingly popular, with more than ten companies producing these sensors commercially [13]. To take advantage of this technology, some researchers have experimented with using only a single PPG waveform for estimating BP. A continuous PPG waveform and one single PPG waveform extracted from it are shown in Figure 1. Shin et al. [14] presented a pressure index (PI) extracted from a single PPG signal to estimate BP. Teng et al. [15] extracted four features of PPG signals to find an optimal feature for BP estimation: width of 2/3 pulse amplitude, width of 1/2 pulse amplitude, systolic upstroke time, and diastolic time. This method established a linear regression model and found that systolic upstroke time and diastolic upstroke time from the PPG wave have higher correlations with BP. However, tests show that such a correlation is not always linear. Gao et al. [16] developed a method for BP estimation using the regression support vector machine (RSVM) method, with RBF kernel and discrete wavelet transform, and obtained better performance.

**Figure 2:**
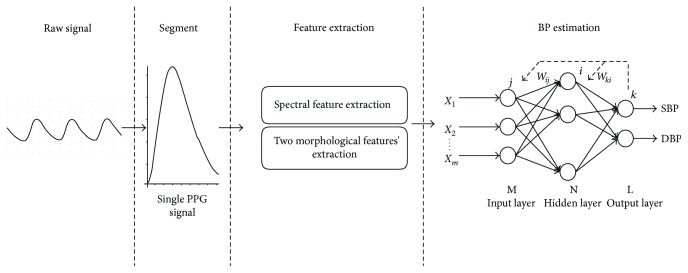
Schematic illustration of presented BP estimation frameworks.

In this paper, a new approach for beat-to-beat BP estimation based on artificial neural networks (ANNs) is presented. Yi et al. [17] have proved that for BP estimation, ANNs have better performance compared to regression analysis using PTT. The presented method uses a multitaper method (MTM) [18] to obtain the spectral components and combines them with two morphological features of a PPG signal, to constitute the input parameters. For wide representation of possible PPG signal and correspondent beat-to-beat BP, we extract the signal from the Multiparameter Intelligent Monitoring in Intensive Care (MIMIC) database [19, 20] for network training and testing. The results show that the presented method achieves better performance using only the PPG signal.  Figure 2 shows the schematic illustration of presented BP estimation frameworks.

The paper is organized as follows: Section 2 describes the overview of the MIMIC database, Section 3 explains the features extracted from a PPG signal and the presentation of the architecture of the ANN, and Section 4 shows the results using different methods. Finally, the conclusion summarizes the paper proposal and briefly anticipates future work.

## 2. Data Description

The MIMIC database is a collection of multiparameter recordings from over 90 ICU patients. The data in each case includes signals and periodic measurements obtained from a bedside monitor, as well as clinical data obtained from the patient's medical record. The recordings vary in length; almost all of them are at least 20 hours, and many are 40 hours or more. In total, the database contains nearly 200 patient-days of real-time signals and accompanying data [21].

**Figure 3:**
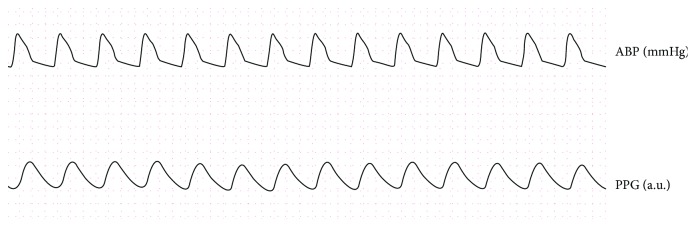
An example record.

The database contains data of ECG (leads I, III, and V), ABP, PAP, PPG, and respiratory signals recorded simultaneously with a 125 Hz sampling rate. In this paper, only the records with both ABP and PPG were extracted.  Figure 3 shows an example record.

In total, there are 58,795 valid intervals of PPG signal (subject number is 72) and corresponding BP values for different people and different time instances. In order to avoid overfitting, we use 70% of them for network training, 15% of them for validation, and 15% of them for testing. The training dataset is presented to the network during training, and the network is adjusted according to its error. The validation dataset is used to measure network generalization and to halt training when generalization stops improving. The test dataset has no effect on training and so provides an independent measure of network performance during and after training. The Levenberg-Marquardt algorithm was chosen for training the ANN. In this algorithm, training automatically stops when generalization stops improving, as indicated by an increase in the mean square error of the validation samples.

## 3. ANN-Based BP Estimation

### 3.1. Multitaper Method

The multitaper method (MTM) [22] takes advantage of an extended version of the spectral representation as follows:

(1) $$\begin{array}{c} x_{t}=\int_{-1/2}^{1/2}e^{-i\omega t}dz\left(t\right). \end{array}$$

In this case, the *x*_*t*_ may contain a number of periodic components in addition to the underlying stationary process as follows:

(2) xt=∑jCjcosωjt+ϕj+ξt=∑jμjeiωtj+μj∗e−iωtj+ξt,

where *ξ*_*t*_ is a zero-mean stationary process with *S*(*f*) not necessarily constant [18]. The above types of processes, called central stationary or conditional stationary processes, are often referred to as having mixed spectra [23]. For these processes, the expected value of the discrete orthogonal increment process dZ(*f*) is no longer zero and can be calculated as follows:

(3) $$\begin{array}{c} E\left\{dZ\left(f\right)\right\}=\sum_{j}\mu_{j}\delta \left(f-f_{j}\right)df, \end{array}$$

where *δ* is the Dirac delta function. The second central moment of dZ(*f*) can be obtained as follows:

(4) $$\begin{array}{c} E\left\{{\left|dZ\left(f\right)-E\left\{dZ\left(f\right)\right\}\right|}^{2}\right\}=S\left(f\right)df. \end{array}$$

For processes with mixed spectra, the first moment of dZ(*f*) gives the deterministic component, while the second central moment of dZ(*f*) gives the continuous nondeterministic component. The classical method has been centered on the estimation of the second moment of dZ(*f*), which gives the continuous component of the spectrum. However, the estimation of the first moment of dZ(*f*) was initially also required. Major opposition to the classical method is predicated on the fact that there is no separation between the deterministic component and nondeterministic component; spurious peaks in the spectrum can be identified as the deterministic component without an objective criterion for differentiating between real and spurious lines [24].

In the MTM spectral estimation, a useful, yet simple, likelihood ratio test for the significance of periodic components is offered by the multiwindow method. This method makes use of multiple data windows, referred to as “Slepian sequences” and “discrete prolate spheroidal sequences.” They are defined as follows:

(5) $$\begin{array}{c} \lambda_{k}\nu_{n}^{\left(k\right)}\left(N,W\right)=\overset{N-1}{\underset{m=0}{\sum }}\frac{\sin\left\{2\pi W\left(n-m\right)\right\}}{\pi \left(n-m\right)}\nu_{m}^{\left(k\right)}\left(N,W\right), \end{array}$$

where *N* is the number of sampling points of a single PPG wave (the sampling rate is 125 Hz), *W* is the spectral bandwidth, and *λ*_*k*_ are the eigenvalues associated with the Slepian sequences *ν*_*n*_^(*k*)^(*N*, *W*), which can be calculated numerically [25]. After Fourier transformation, the Slepian functions can be calculated as follows:

(6) $$\begin{array}{c} v_{k}\left(f\right)=\overset{N-1}{\underset{n=0}{\sum }}v_{n}^{\left(k\right)}\left(N,W\right)e^{-i2\pi fn}. \end{array}$$

In the interval (*f* − *W*, *f* + *W*), the energy concentration of the above Slepian functions is maximum. Furthermore, the bias from all frequencies is remote from the frequency of the window width times the number of observations, and thus the use of these sequences is very effective in eliminating window leakage [26].

The MTM calculates the expansion or eigen coefficients of input *X*_*t*_ as a first step as follows:

(7) $$\begin{array}{c} y_{k}\left(f\right)=\overset{N-1}{\underset{t=0}{\sum }}x_{t}\nu_{i}^{\left(k\right)}\left(N,W\right)e^{-i2\pi ft}. \end{array}$$

Combining the above equations, the expected value of *y*_*k*_(*f*) can be obtained as follows:

(8) $$\begin{array}{c} E\left\{y_{k}\left(f\right)\right\}=\mu V_{k}\left(f-f_{0}\right)+\mu^{*}V_{k}\left(f+f_{0}\right). \end{array}$$

At *f* = *f*_0_,

(9) $$\begin{array}{c} E\left\{y_{k}\left(f_{0}\right)\right\}=\mu V_{k}\left(0\right)+\mu^{*}V_{k}\left(2f_{0}\right)\approx \mu V_{k}\left(0\right), \end{array}$$

assuming 2*f*_0_ > *W* and thus neglecting the second term in (9), since *V*_*k*_ is highly concentrated in the interval (*f* − *W*, *f* + *W*).

By minimizing the residual local squared error, that is, when *f* = *f*_0_, the *μ* can be estimated. The squared error can be described as follows:

(10) $$\begin{array}{c} e^{2}\left(\mu ,f\right)=\overset{N-1}{\underset{k=0}{\sum }}{\left|y_{k}\left(f\right)-\mu \left(f\right)V_{k}\left(0\right)\right|}^{2}. \end{array}$$

The result is given as follows:

(11) $$\begin{array}{c} \hat{\mu }\left(f\right)=\frac{\sum_{k=0}^{K-1}V_{k}^{*}\left(0\right)y_{k}\left(f\right)}{\sum_{k=0}^{K-1}{\left|V_{k}\left(0\right)\right|}^{2}}. \end{array}$$

An *F* test can be used to test for the significance of a line component at *f*, and the location of its maximum value provides an estimation of the line frequency.

In this paper, the periods of interest are nearly as long as the data. Thus, a line component at zero frequency is included and the estimation of *μ*(0) from (11) is used as an alternative estimation of the mean, which will result in the preservation of the continuous part of the spectrum at zero frequency [23]. The continuous part of the spectrum can be calculated as follows:

(12) $$\begin{array}{c} \hat{S}\left(f\right)=\frac{1}{K}\overset{K-1}{\underset{k=0}{\sum }}{\left|y_{k}\left(f\right)\right|}^{2}. \end{array}$$

However, according to (4), the spectrum near a significant line component with frequency *f*_0_ must be reconstructed, by subtracting the contribution of the line component as follows [27]:

(13) $$\begin{array}{c} {\hat{S}}_{r}\left(f\right)=\frac{1}{K}\overset{K-1}{\underset{k=0}{\sum }}{\left|y_{k}\left(f\right)-\hat{\mu }\left(f_{0}\right)V_{k}\left(f-f_{0}\right)\right|}^{2}. \end{array}$$

### 3.2. Feature Extraction

Several spectral and morphological features are used to characterize the single PPG signal. The systolic upstroke time (ST) and diastolic time (DT) presented in [15] are used as the two morphological features. Then the MTM is used to extract the spectral features.

**Figure 4:**
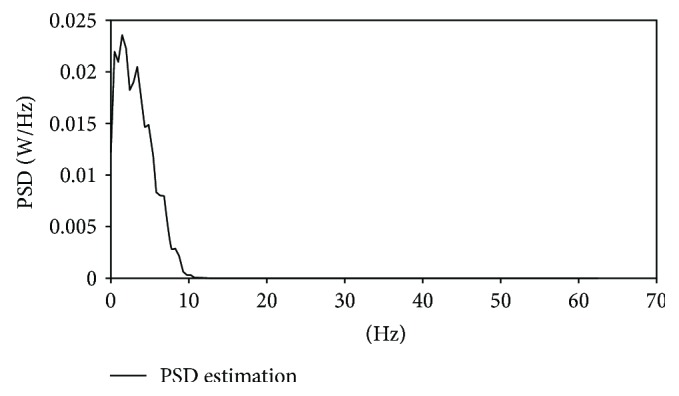
Frequency of single PPG signal.

As shown in Figure 4, the dominant frequency of single PPG waveform is mostly focused in the interval of low frequency (0.1~10 Hz).

**Figure 5:**
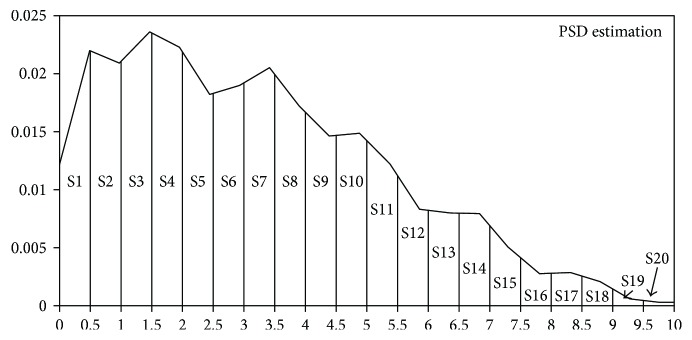
Spectral features extracted from single PPG signal.

As a result, this method tries to extract the spectral character in the interval of low frequency (0.1~10 Hz). We calculate the power of every 0.5, 1, 2, 5, and 10 Hz interval. Then, we use them as input separately to determine the optimal number of input parameters, and the results are as listed in Table 2 of Section 4. The best results are obtained when the interval is 0.5 Hz, as presented in Figure 5.

In total, the 22 parameters, including the times of systolic and diastolic parts and spectral features, are used to train the ANN.

### 3.3. Artificial Neural Network Architecture

There are various ANN architectures for fitting the input data to target, such as counter propagation, learning vector quantization, and radial basis function. Despite good performance, these architectures require large numbers of neurons and cannot be applied in the case of a big training set, due to their substantial memory requirements.

**Figure 6:**
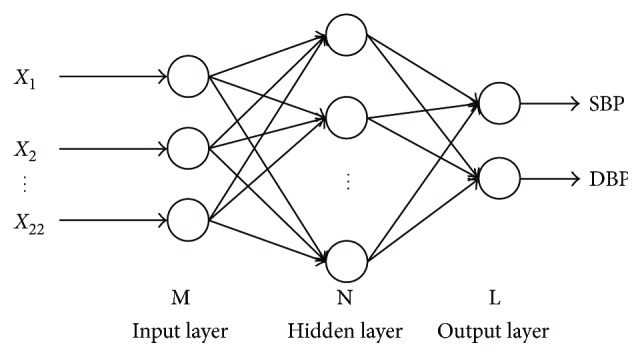
The architecture of ANN.

In this paper, PPG features are fed to a multilayer perceptron architecture, which has 22 input neurons (the number of input parameters, as mentioned above) and 2 output neurons, to simultaneously estimate SBP and DBP. This architecture is shown in Figure 6.

## 4. Experimental Results and Discussion

**Figure 7:**
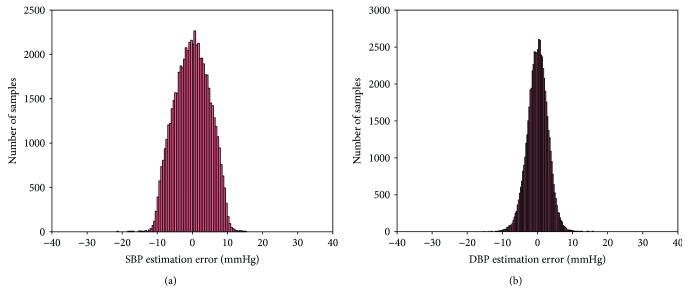
Histograms of error: (a) SBP estimation error and (b) DBP estimation error.

Figure 7 shows the histograms of the errors, calculated as the difference between real SBP/DBP and the output of the ANN, for the proposed method. The mean difference and standard deviation between the estimated BP and measured BP are −0.0217 ± 4.8950 mmHg for SBP and 0.0975 ± 2.9160 mmHg for DBP.

To further evaluate the performance of the presented method, other two BP [15, 16] estimation methods are chosen for comparison in this paper.  Table 3 lists the number of subjects in the above studies.

As indicated earlier, performance is assessed on effective records from the MIMIC database and is processed in a MATLAB (MathWorks, Natick, MA, USA) environment. Absolute error *e* and relative error *e*_r_ are used to evaluate the performance, which are, respectively, defined as

(14) $$\begin{array}{c} e={BP}_{estimated}-BP,\\ e_{r}=\frac{{BP}_{estimated}-BP}{BP}. \end{array}$$

Table 2 lists the performance results of different numbers of input parameters, and Table 4 lists the results of performance on the test database for the linear regression, RSVM-based method, and ANN-based method with different feature extraction techniques. The results are presented as mean and standard deviation of absolute error *e* and relative error *e*_r_, among reference SBP/DBP and estimated values. For evaluation of the performance of this model in a single individual, test data is divided into individual subsets by index provided by PhysioNet [21] and is used to test performance on each subset, respectively. The estimated performance of the method applied to single individuals is listed in Table 5.

Compared with the other methods, our method has better performance and can be confidently said to provide an effective detection technique for wearable devices and mobile software in the field of hypertension.

## 5. Conclusion and Future Work

In this study, we propose a noninvasive and beat-to-beat method of BP estimation determined only from PPG signal. The MTM is used to extract representative features to improve precision and velocity. This is achieved using a typical-structure feed forward ANN. With the wearable PPG sensor becoming an increasingly popular technology, this method has practical significance as part of a big data solution.

According to the Association for the Advancement of Medical Instrumentation (AAMI), the mean and deviation absolute error between the device and the mercury standard sphygmomanometer can be larger than 5 ± 8 mmHg as well as the number of simultaneous readings agrees within 10 mmHg for 95% or more of the recordings and within 5 mmHg for 85% or more of simultaneous observations [13, 28]. Our future research will investigate how to improve the efficiency of the estimation algorithm, especially in a single individual. We will combine the method with data such as patient age, sex, and previous medical disorders. Hybrid and adaptable methods will also be considered.

## Figures and Tables

**Table 1:** Classification of hypertension in adults (age > 18 years).

Blood pressure classification	BP (mmHg)	
	Systolic	Diastolic
Normal	<120	And <80
Prehypertension	120–139	Or 80–89
Stage 1 hypertension	140–159	Or 90–99
Stage 2 hypertension	≥160	Or ≥100

**Table 2:** Performance results of different number of the input parameters.

Number of the input parameters	SBP		DBP	
	*e* (mmHg)	*e*_r_ (%)	*e* (mmHg)	*e*_r_ (%)
3 parameters (10 Hz interval)	8.29 ± 6.60	6.00 ± 5.28	6.19 ± 6.63	9.14 ± 7.45
4 parameters (5 Hz interval)	7.62 ± 6.08	5.49 ± 4.77	4.31 ± 3.83	3.84 ± 3.39
7 parameters (2 Hz interval)	6.59 ± 5.25	4.72 ± 4.02	4.34 ± 5.23	4.72 ± 3.91
12 parameters (1 Hz interval)	4.64 ± 3.63	3.42 ± 2.61	3.69 ± 2.74	3.12 ± 4.11
22 parameters (0.5 Hz interval)	4.02 ± 2.79	2.84 ± 2.00	2.27 ± 1.82	4.39 ± 3.60

**Table 3:** Number of subjects in different methods.

Method	Number of subjects
Linear regression [15]	15
RSVM based [16]	65
Proposed method	72

**Table 4:** Performance results of the linear regression, SVRM-based method, and ANN-based method with different feature extraction technique on the test database.

Method	Number of input parameters	SBP		DBP	
		*e* (mmHg)	*e*_r_ (%)	*e* (mmHg)	*e*_r_ (%)
Linear regression [15]	2	9.80 ± 8.09	8.94 ± 7.57	5.88 ± 5.11	10.26 ± 8.83
RSVM based [16]	13 for SBP 22 for DBP	5.07 ± 4.84	4.32 ± 3.59	4.31 ± 3.83	3.84 ± 3.39
ANN based (this method)	22	4.02 ± 2.79	2.84 ± 2.00	2.27 ± 1.82	4.39 ± 3.60

**Table 5:** The performance results based on individual measurements.

Record	SBP		DBP	
	*e* (mmHg)	*e*_r_ (%)	*e* (mmHg)	*e*_r_ (%)
Subject number 211 (F, 67)	4.68 ± 4.05	4.64 ± 4.62	2.60 ± 1.59	4.49 ± 2.70
Subject number 212 (M, 84)	5.10 ± 4.32	3.65 ± 3.25	2.95 ± 1.98	5.44 ± 3.88
Subject number 213 (F, 82)	3.03 ± 2.71	3.00 ± 2.56	3.12 ± 1.98	5.50 ± 3.43
Subject number 214 (F, 72)	4.10 ± 2.72	2.91 ± 1.92	2.50 ± 1.64	5.48 ± 3.65
Subject number 216 (M, 67)	3.35 ± 2.32	3.28 ± 2.29	1.91 ± 1.58	4.52 ± 3.33
Subject number 224 (M, 21)	2.89 ± 2.15	2.37 ± 1.82	2.16 ± 1.59	4.12 ± 3.04
Subject number 225 (M, 73)	4.51 ± 2.79	3.79 ± 2.39	2.08 ± 1.52	4.92 ± 3.64
Subject number 226 (M, 68)	4.44 ± 2.81	3.49 ± 2.20	2.32 ± 1.70	4.18 ± 3.50
Subject number 230 (F, 75)	5.18 ± 3.63	3.24 ± 2.28	1.82 ± 1.42	4.66 ± 3.51
Subject number 235 (F, 67)	3.55 ± 2.59	2.47 ± 1.80	2.09 ± 1.39	3.81 ± 2.98
Subject number 237 (F, 63)	6.14 ± 4.29	4.10 ± 2.68	2.92 ± 2.06	4.14 ± 2.69
Subject number 240 (M, 68)	4.55 ± 3.26	3.65 ± 2.56	1.84 ± 1.76	3.87 ± 3.09
Subject number 241 (F, 76)	3.95 ± 2.59	3.23 ± 2.20	3.45 ± 2.19	5.71 ± 3.97
Subject number 243 (M, 90)	5.21 ± 3.28	4.41 ± 2.78	2.92 ± 1.88	4.99 ± 3.15
Subject number 245 (F, 63)	4.03 ± 2.84	3.01 ± 2.12	3.40 ± 2.12	5.77 ± 3.65
Subject number 252 (M, 52)	6.08 ± 2.96	5.30 ± 2.68	2.70 ± 2.07	5.58 ± 3.42
Subject number 259 (F, 76)	4.57 ± 3.07	3.45 ± 2.33	3.26 ± 2.10	4.51 ± 3.34
Subject number 262 (F, 65)	5.15 ± 3.60	4.36 ± 3.15	2.77 ± 1.93	5.36 ± 3.43
Subject number 264 (M, 65)	4.11 ± 2.66	3.14 ± 2.04	2.79 ± 1.91	4.64 ± 3.21
Subject number 267 (M, 67)	3.72 ± 2.56	2.84 ± 1.94	2.56 ± 1.82	4.66 ± 3.06
Subject number 269 (F, 82)	4.34 ± 2.95	3.26 ± 2.22	3.39 ± 2.20	4.28 ± 2.91
Subject number 276 (F, 66)	3.76 ± 2.53	3.11 ± 2.13	2.78 ± 1.95	4.01 ± 3.07
Subject number 277 (F, 71)	4.33 ± 2.85	3.51 ± 2.31	2.85 ± 2.00	3.94 ± 2.74
Subject number 279 (F, 85)	4.15 ± 2.65	3.40 ± 2.21	3.03 ± 2.06	4.13 ± 2.90
Subject number 280 (M, 60)	4.50±2.84	3.93 ± 2.54	3.57 ± 2.52	4.06 ± 2.78
Subject number 281 (M, 61)	5.02±3.58	4.35 ± 3.19	2.70 ± 1.83	5.16 ± 3.53
Subject number 284 (F, 59)	4.73 ± 3.19	3.37 ± 2.37	3.15 ± 2.16	4.96 ± 3.45
Subject number 285 (M, 55)	5.70 ± 4.73	4.11 ± 4.04	2.66 ± 1.97	5.12 ± 3.42
Subject number 286 (F, 34)	4.30 ± 3.01	3.59 ± 2.52	2.86 ± 1.82	4.40 ± 2.77
Subject number 288 (F, 59)	4.84 ± 3.00	3.70 ± 2.33	3.27 ± 2.01	5.40 ± 4.28
Subject number 289 (F, 61)	5.10 ± 3.77	4.67 ± 3.49	2.85 ± 1.83	6.12 ± 4.38
Subject number 293 (F, 71)	4.44 ± 3.82	3.86 ± 2.51	2.92 ± 2.12	4.20 ± 2.98
Subject number 401 (F, 64)	7.11 ± 5.64	5.54 ± 4.58	3.97 ± 2.91	4.32 ± 3.27
Subject number 404 (F, 87)	4.01±3.78	2.73 ± 1.91	2.07 ± 2.73	5.03 ± 3.38
Subject number 408 (M, 45)	4.10 ± 2.70	2.88 ± 1.89	3.01 ± 2.01	6.08 ± 4.20
Subject number 410 (M, 57)	3.53 ± 3.48	3.10 ± 3.15	3.18 ± 2.13	5.69 ± 3.56
Subject number 411 (F, 82)	3.40 ± 3.33	3.42 ± 2.38	3.45 ± 2.36	5.07 ± 3.41
Subject number 415 (F, 54)	3.06 ± 2.03	2.99 ± 2.00	2.66 ± 1.97	4.71 ± 3.39
Subject number 417 (M, 86)	3.13 ± 2.14	2.95 ± 2.03	3.74 ± 2.08	7.10 ± 3.97
Subject number 418 (M, 52)	3.59±3.41	3.54 ± 2.39	2.65 ± 1.86	5.74 ± 3.77
Subject number 422 (F, 84)	3.97 ± 3.29	4.03 ± 4.40	3.18 ± 2.13	4.30 ± 3.09
Subject number 430 (M, 91)	2.68 ± 2.06	2.61 ± 2.00	3.62 ± 2.35	5.52 ± 4.04
Subject number 434 (F, 52)	6.54 ± 6.35	4.30 ± 4.38	3.29 ± 2.00	4.32 ± 4.11
Subject number 436 (F, 87)	7.59 ± 6.80	4.92 ± 4.61	2.97 ± 1.91	5.19 ± 4.75
Subject number 437 (M, 75)	8.36 ± 7.79	5.57 ± 5.37	2.89 ± 2.04	4.41 ± 3.18
Subject number 438 (M, 78)	3.73 ± 2.67	3.48 ± 2.50	2.31 ± 1.64	3.89 ± 2.86
Subject number 439 (F, 75)	4.44 ± 3.87	3.60 ± 2.30	2.14 ± 1.51	5.85 ± 3.58
Subject number 443 (M, 75)	4.52 ± 3.75	3.82 ± 3.36	2.34 ± 1.66	4.82 ± 3.48
Subject number 446 (M, 73)	4.97 ± 4.90	3.91 ± 2.31	2.09 ± 1.51	5.82 ± 3.67
Subject number 447 (M, 50)	4.01 ± 3.65	3.32 ± 2.21	3.76 ± 2.35	4.13 ± 2.92
Subject number 449 (M, 75)	3.93 ± 3.74	2.91 ± 2.01	3.45 ± 2.23	5.69 ± 3.70
Subject number 450 (F, 76)	4.73 ± 3.69	3.21 ± 2.39	3.53 ± 2.27	4.66 ± 3.22
Subject number 452 (M, 73)	4.34 ± 3.73	3.61 ± 3.28	2.90 ± 2.13	6.73 ± 3.95
Subject number 455 (M, 49)	4.19 ± 3.69	3.48 ± ± 2.18	2.16 ± 2.96	5.47 ± 3.41
Subject number 456 (M, 84)	3.69 ± 3.52	2.77 ± 1.89	3.67±2.13	5.38 ± 3.89
Subject number 457 (F, 80)	4.28 ± 3.91	3.36 ± 2.39	2.52 ± 1.81	4.47 ± 3.41
Subject number 458 (F, 73)	3.87 ± 2.85	3.32 ± 2.48	2.40 ± 1.72	5.83 ± 3.96
Subject number 464 (F, 49)	4.05 ± 2.86	3.43 ± 2.51	2.58 ± 1.79	5.32 ± 3.63
Subject number 466 (M, 70)	4.42 ± 3.55	3.69 ± 2.18	2.10 ± 1.92	6.73 ± 3.95
Subject number 468 (F, 76)	3.49 ± 2.32	2.95 ± 1.98	2.63 ± 1.89	5.47 ± 3.41
Subject number 471 (F, 78)	3.72 ± 2.57	3.16 ± 2.21	3.99 ± 2.25	5.83 ± 3.39
Subject number 472 (M, 79)	3.29 ± 2.21	2.66 ± 2.84	3.30 ± 2.06	4.78 ± 3.60
Subject number 474 (M, 75)	5.04 ± 3.62	4.20 ± 4.06	3.64 ± 2.49	5.12 ± 3.67
Subject number 476 (F, 72)	4.20 ± 3.72	3.48 ± 4.18	3.24 ± 2.34	4.47 ± 3.45
Subject number 477 (M, 67)	4.07 ± 3.68	3.17 ± 2.80	3.17 ± 2.08	4.12 ± 3.67
Subject number 478 (M, age not recorded)	4.44 ± 2.79	3.82 ± 2.37	2.95 ± 1.99	5.93 ± 4.12
Subject number 479 (F, 77)	4.78 ± 4.04	3.43 ± 3.91	3.23 ± 2.08	4.96 ± 4.71
Subject number 480 (M, 52)	5.20 ± 4.81	4.25 ± 3.99	3.17 ± 2.08	5.18 ± 3.78
Subject number 481 (F, 73)	2.91 ± 3.76	3.55 ± 4.01	2.76 ± 1.94	5.44 ± 3.81
Subject number 482 (F, 92)	3.88 ± 2.67	3.34 ± 2.44	2.51 ± 1.83	4.47 ± 3.45
Subject number 484 (M, 60)	4.52 ± 2.74	3.81 ± 4.74	2.82 ± 2.00	4.94 ± 4.12
Subject number 485 (M, 69)	4.73 ± 3.89	3.54 ± 2.50	2.34 ± 1.74	4.18 ± 3.68
Subject number 488 (age and gender not recorded)	4.04 ± 2.75	3.24 ± 2.24	3.12 ± 2.16	4.92 ± 3.24
